# Association of preterm birth and congenital anomalies: findings of a Brazilian multicenter study

**DOI:** 10.61622/rbgo/2026rbgo35

**Published:** 2026-07-17

**Authors:** Marcelo Luis Nomura, Luiz Carlos Santos-Junior, Jose Paulo Siqueira Guida, Tabata Zumpano Santos, Giuliane Jesus Lajos, Rodolfo Carvalho Pacagnella, Patricia Moretti Rheder, Ricardo Porto Tedesco, Renato Teixeira Souza, Maria Laura Costa, Renato Passini-Junior, Jose Guilherme Cecatti

**Affiliations:** 1 Universidade Estadual de Campinas Faculdade de Ciências Médicas Departamento de Obstetrícia e Ginecologia Brazil Departamento de Obstetrícia e Ginecologia, Faculdade de Ciências Médicas, Universidade Estadual de Campinas, Brazil; 2 Faculdade de Medicina de Jundiaí Departamento de Obstetrícia e Ginecologia Jundiaí SP Brazil Departamento de Obstetrícia e Ginecologia, Faculdade de Medicina de Jundiaí, Jundiaí, SP, Brazil

**Keywords:** Congenital abnormalities, Premature birth, Term birth, Infant, newborn, Infant mortality, Gestational age, Maternal age

## Abstract

**Objective:**

to analyze the prevalence of congenital anomalies among women who had preterm births, their association with gestational age, characteristics of pregnancy termination and perinatal outcomes.

**Methods:**

A secondary analysis of the Brazilian Multicenter Study on Preterm Birth (EMIP), a multicenter cross-sectional study in 20 maternity hospitals, comparing cases of preterm birth with and without anomalies. A random sample of term births served as controls. The risk of malformations was estimated by maternal age, gestational age, type of onset of labour, mode of delivery, and neonatal outcomes using OR (95%CI) for categorical variables.

**Results:**

Among 4,150 preterm births and 1,146 selected term controls, congenital anomalies were found in 7.21% of cases (8.36% in preterm vs. 3.1% in term). Preterm birth increased the risk of anomalies by 2.89-fold (95% CI: 2.03–4.13). Cardiovascular anomalies were most common (19.1%), followed by central nervous system anomalies (18.8%) and abdominal wall defects (12.5%). The highest risk was observed at 28–31 weeks (OR 4.57, 95%CI 3.01–6.94). Elective cesarean delivery was more frequent among neonates with abnormalities (40.1% vs. 32.1%, OR 1.41, 95% CI: 1.13–1.77). Neonates with abnormalities had a higher risk of low 5^th^ minute Apgar scores (OR 2.68, 95% CI: 2.01–3.56), birth weight <1.5 kg (OR 1.46, 95% CI: 1.14–1.87), and adverse outcomes such as NICU admission, sepsis, and neonatal mortality (OR 6.0, 95% CI: 4.58–7.85).

**Conclusion:**

Prematurity is associated with congenital anomalies, leading to increased neonatal morbidity, mortality, and admission to NICU.

## Introduction

Preterm birth is one of the major global challenges in public health. Data from the World Health Organization (WHO) estimate that approximately 15 million children are born preterm each year worldwide. According to the WHO, a newborn is considered preterm if born before 37 weeks of gestation. Preterm births are further classified as extremely preterm (born before 28 weeks), very preterm (born between 28–34 weeks), and late preterm (born after 34 weeks). Most preterm births fall into the late preterm category, accounting for approximately 85% of all preterm deliveries.^(1)^

Currently, an increase in preterm birth rates is observed, influenced by multiple factors, including a rise in multiple pregnancies due to the widespread use of assisted reproductive technologies,^(1)^ and a higher prevalence of pregnancies in women with comorbidities such as hypertension, diabetes mellitus, obesity, among others. These conditions together with fetal anomalies have been associated with preterm birth in several studies.^(2-4)^ In addition, it seems to occur a general improvement in reporting and detecting preterm births, especially in low and middle-income countries.^(5)^

Although preterm birth accounts for approximately 10% of deliveries worldwide, it is responsible for 35% of deaths within the first year of life. It is the leading cause of early neonatal mortality and the second leading cause of childhood mortality up to five years of age. According to the WHO, one million deaths per year are related to complications of preterm birth.^(5)^

A congenital anomaly is a structural, functional, or metabolic abnormality present at birth, affecting the body's appearance and/or function. The incidence of congenital anomalies is estimated at 3% of all births in the United States. Data from the ECLAMC (Latin America Collaborative Study of Congenital Malformations) show that the prevalence of anomalies in Brazil was 4% among live births and 14.8% among stillbirths.^(6)^ There is a significant association between preterm birth and congenital anomalies, either due to shared risk factors or because certain anomalies necessitate medical interventions possibly leading to preterm delivery.^(7)^

Some studies indicate that the prevalence of congenital anomalies among preterm infants is 18%, with anencephaly, gastroschisis, gastrointestinal atresia, hydrocephalus, and renal agenesis being the most commonly reported anomalies. Others, such as congenital heart defects and neural tube closure defects, were also significant. It has been observed that neonates with congenital anomalies have a higher probability of being born before 32 weeks of gestation and with a birth weight below 1.5 kg. Overall mortality due to complications of congenital anomalies increases tenfold when they are associated with preterm birth.^(8)^

There is a lack of comprehensive data on the association between congenital anomalies and preterm birth in Brazil. This study aims to analyze the prevalence of congenital anomalies among women who had preterm births in Brazil, its association with gestational age, characteristics of pregnancy termination and perinatal outcomes.

## Methods

This is a secondary analysis of the Brazilian Multicenter Study on Preterm Birth (EMIP). Its main findings, as well as the research protocol and methodological aspects of its implementation, have been previously published elsewhere.^(9-11)^ The EMIP collected data from 20 Brazilian secondary or tertiary maternity hospitals distributed across three country regions during 16 months from 2011 to 2013.

The EMIP was mainly a cross-sectional study in which data were collected from all preterm births occurring at the participating centres during the study period. Additionally, a random sample of cases of term births occurring during the same period at the same facilities was included to serve as controls for the case-control component of the study as originally planned. The selection of these controls was performed as planned, including the first childbirth at term occurring just after the preterm birth included in the same health facility, until completing the estimated sample of controls. The original hypothesis that justifies the current analysis is that the prevalence of congenital anomalies is still high among pregnancies in Brazil, especially when a preterm birth occurs.

The study received ethical approval before data collection started. It was approved by the Research Ethics Committee of the University of Campinas (Protocol #704/2009) that was the coordinating centre, and subsequently by the ethics committees of each additional participating centre. Participants were included only after obtaining a signed informed consent.

After enrollment, data collection was conducted through participant interviews and a review of medical records and antenatal care cards performed by researchers specifically trained for the study. The data were stored in an internet-based platform under the responsibility of the coordinating centre. Information was gathered on maternal characteristics, clinical follow-up during pregnancy, gestational conditions, termination of pregnancy, and perinatal outcomes.

For the assessment of congenital anomalies, diagnoses established before birth - obtained through antenatal and ultrasound follow-ups — were considered. Additionally, data on anomalies diagnosed after birth were collected through postnatal clinical evaluation, with follow-up of the included neonates up to 60 days after delivery.

For this analysis, preterm and term birth participants were included to estimate the prevalence of and estimated risks for congenital anomalies across different maternal age and gestational age groups. Anomalies were classified according to the affected system and categorised into cardiovascular, central nervous system, abdominal wall closure defects, musculoskeletal, genitourinary, chromosomal defects, facial malformations, gastrointestinal, thoracic, hydrops-related, skin-related, and other anomalies. When observed in isolation, the presence of a patent foramen *ovale* or persistent *ductus arteriosus* was not considered an anomaly, as they may result from the prematurity itself.

Subsequently, all neonates were selected and divided into two groups of exposure: one consisting of neonates with congenital anomalies and another without anomalies for comparison. These groups were compared for estimating the risk of anomaly using odds ratios (OR) with the corresponding 95% confidence intervals (CI) for categories of maternal age and gestational age. Then, the risks of different types of onset of labour, mode of delivery and adverse neonatal outcomes were also estimated for gestations with only preterm fetuses with anomalies using OR (95%CI) for categorical variables. Considering the main objective of a more descriptive approach for the impact of these cases of preterm fetuses with congenital anomalies, no additional adjusted analyses were performed. A p-value <0.05 was considered significant. Statistical analyses were conducted using EpiInfo 7.2 software.

## Results

A total of 4,150 women were included in the preterm birth group and 1,146 in the term birth control group. Congenital anomalies were observed in 382 of the total cases (7.21%), with 347 cases among preterm births (8.36% of this group) and 35 cases among the control sample of term births (3.1% of this group). Regarding the causal conditions of preterm birth, the distribution among neonates with anomalies was similar to that without: 34% due to spontaneous preterm labour (n=118), 29.4% due to preterm premature rupture of membranes (n=102), and 36.60% (n=127) due to medically indicated preterm birth or provider-initiated preterm birth (data not shown in tables). Regarding the type of anomaly, the most frequent in this study were cardiovascular anomalies (19.1%), central nervous system anomalies (18.8%), abdominal wall defects (12.5%), musculoskeletal (12.3%) and genitourinary tract anomalies (11.2%). Other categories included chromosomal defects (7.6%), craniofacial/cervical anomalies (7.0%), and gastrointestinal tract defects (5.2%). The prevalences of the identified congenital anomalies among fetuses with anomalies and among all births are presented in table 1.

**Table 1:** Prevalence of types of malformations (MF) in a multicenter study of preterm birth (PTB) in Brazil

Classification of MF	n	Prevalence among all births with MF (n=382) %	Prevalence among all births (n=5296) %
Cardiovascular	73	19.1	1.3
Central nervous system	72	18.8	1.3
Abdomen	48	12.5	0.9
Musculoskeletal	47	12.3	0.8
Genito-urinary tract	43	11.2	0,8
Chromosomic defects	29	7.6	0.5
Eye, face, neck and ear	27	7.0	0.5
Gastrointestinal	20	5.2	0.3
Respiratory and thoracic	7	1.8	0.1
Hydrops	6	1.5	0.1
Skin	2	0.5	0.03
Others	8	2.1	0.1
Total	382	100	7.21

Table 2 presents the frequency of births according to different maternal age and gestational age ranges, comparing neonates with anomalies to those without. The upper group of maternal age, with 40 years old or more, showed a higher estimated risk of fetal anomaly (OR 1.69, 95%CI 1.07-2.65). A higher estimated risk of anomalies was also observed for groups of gestational ages between 22 and 27 weeks (4.97 vs 5.88%, OR 2.09, 95%CI 1.18-3.70), for 28 to 31 weeks (18.85% vs. 10.18%, OR 4.57, 95% CI: 3.01–6.94) and between 32 and 36 weeks (67.02% vs. 61.33%, OR 2.69, 95% CI: 1.88–3.86) comparatively with those born at term. Altogether, for any preterm birth, the risk of anomaly was estimated to be 2.89 times higher than for term births (95% CI: 2.03–4.13). The presence of polyhydramnios was nearly 10 times higher in the preterm with anomaly group compared to preterm neonates without (OR 9.26, 95%CI: 6.35–13.51), with 15.9% of neonates with anomaly presenting polyhydramnios (data not shown in tables).

**Table 2:** Estimated risk of malformation (MF) among all neonates according to maternal age and gestational age groups in a multicenter study in Brazil

Characteristics	All neonates with MF n(%)	All neonates witho ut MF n(%)	OR	95%CI
**Maternal age (years)**				
<30	262(68.6)	3384(68.9)	1.0	Ref.
30-34	63(16.5)	859(17.5)	0.95	0.71-1.26
35-39	34(8.9)	495(10.1)	0.89	0.61-1.28
≥ 40	23(6.0)	176(3.6)	**1.69**	**1.07-2.65**
**Gestational Age (weeks)**				
22-27	19(4.97)	289(5.88)	**2.09**	**1.18 – 3.70**
28-31	72(18.85)	500(10.18)	**4.57**	**3.01 – 6.94**
32-36	256(67.02)	3014(61.33)	**2.69**	**1.88– 3.86**
All preterm (22-36)	347(90.84)	3803 (77.39)	**2.89**	**2.03 – 4.13**
37-42	35(9.16)	1111(22.61)	1.0	Ref.
Total	382	4914		

Values in bold mean they are significant at p<0.05

Regarding the type of onset of labour, table 3 shows that 40.1% of neonates with anomaly were delivered by elective cesarean section, compared to 32.1% among those without anomaly (OR 1.41, 95%CI: 1.13–1.77). When elective cesarean sections were combined with cesarean sections performed during labour, the total cesarean section rate among neonates with anomaly reached 66.3% (OR 1.9, 95%CI: 1.51–2.39) in comparison with 50.8% among those without anomaly. The main indications for cesarean section among fetuses with anomaly as recorded in medical charts, were fetal distress (29.5%), the congenital anomaly itself (29.5%), breech presentation (19.7%), and maternal hypertension (13.8%). In 48 cases (18.9%), the presence of congenital anomaly was listed as the sole indication, for cesarean delivery, mainly for conditions probably compromising the occurrence of a vaginal delivery, including oversize of the fetus or some parts of its body as was the case for hydrocephalus, conjoined twins, and sacrococcygeal teratoma. Neonates with anomaly also had statistically lower rates of labor induction (OR 0.57, 95%CI: 0.38–0.83) and vaginal delivery (OR 0.52, 95%CI: 0.41–0.65). A total of 10.9% of multiple gestations were observed among neonates with anomaly and 10.4% among those without, a non-significant difference (p=0.31, data not shown).

**Table 3:** Estimated risks of type of onset of labor and mode of delivery of preterm births (PTB) according to fetal malformation in a multicenter study in Brazil

Onset of labor		PTB with MF (n=347) n(%)	PTB without MF (n=3803) n(%)	OR*	95%CI
Spontaneous onset		178(51.3)	2039(53.6)	0.91	0.73-1.14
Labor induction		30(8.6)	544(14.3)	**0.57**	**0.38-0.83**
Elective C-section		139(40.1)	1220(32.1)	**1.41**	**1.13-1.77**
Mode of delivery					
	Vaginal birth	108(31.1)	1773(46.6)	**0.52**	**0.41-0.65**
	Forceps	4(1.2)	47(1.2)	0.93	0.33-2.60
	Cesarean section	230(66.3)	1934(50.8)	**1.90**	**1.51-2.39**
	Vaginal+cesarean	5(1.4)	49(1.3)	1.12	0.44-2.83

* Reference for each category of onset of labor and mode of delivery is the complement for the total numbers in each group of exposure. Values in bold mean they are significant at p<0.05

Table 4 shows adverse birth outcomes where neonates with anomaly had a 2.6-fold higher risk of having an Apgar score <7 at both the 1^st^ (OR 2.62, 95%CI: 2.09–3.27) and 5^th^ minutes (OR 2.68, 95%CI: 2.02–3.56). Neonates with anomaly were 46% more likely to be born with a birth weight below 1.5 kg (OR 1.46, 95%CI: 1.14–1.87). No significant difference was found for birth weights <2.5 kg or <1 kg. Neonates with anomaly had also a higher estimated risk of NICU admission (OR 2.59, 95%CI 2.06-3.25), and lower hospital discharge rates before 60 days (OR 3.29, 95%CI: 1.87–5.81). Moreover, they had a sixfold higher neonatal mortality rate compared to those without anomaly (OR 6.0, 95%CI: 4.59–7.85). Even more, neonates with anomaly were twice as likely to develop sepsis, whether based on clinical parameters (OR 2.08, 95%CI: 1.61–2.70) or positive blood cultures (OR 2.27, 95%CI: 1.48–3.49) (Table 4).

**Table 4:** Estimated risks of adverse neonatal outcomes of preterm births (PTB) according to malformation in a multicenter study

Neonatal outcomes	PTB with MF (n 347) n(%)	PTB without MF (n 3803) n(%)	OR (p-value)	95%CI
Birth weight <2.5 kg	258(74.35)	2701(71.02)	1.18	0.92-1.52
Birth weight <1.5 kg	94(27.09)	771(20.27)	**1.46**	**1.14-1.87**
Birth weight <1 kg	28(8.07)	349(9.18)	0.87	0.58-1.29
Apgar 1^st^ min <7	159(45.82)	929(24.43)	**2.62**	**2.09-3.27**
Apgar 5^th^ min <7	71(20.46)	333(8.76)	**2.68**	**2.02-3.56**
Admission to NICU	221(63.7)	1536(40.4)	**2.59**	**2.06-3.25**
Clinical neonatal sepsis	87(25.07)	526(13.83)	**2.08**	**1.61-2.70**
Neonatal sepsis (hemoculture +)	27(7.78)	136(3.58)	**2.27**	**1.48-3.49**
Neonatal death	98(28.24)	234(6.15)	**6.00**	**4.59-7.85**
Still admitted (60 days)	16(4.61)	55(1.45)	**3.29**	**1.87-5.81**

Reference for each category of onset of labor and mode of delivery is the complement for the total numbers in each group of exposure. Values in bold mean they are significant at p<0.05

## Discussion

This study evaluated data from a Brazilian multicenter study on prematurity, the EMIP, and found that among a total of 5,296 births, the global prevalence of congenital anomalies was 7.21%, with a significantly higher rate among preterm births. The most frequent anomalies were those affecting the cardiovascular system, central nervous system, and abdominal wall. It was observed that premature newborns with anomalies had a higher likelihood of low Apgar score, neonatal sepsis and admission to a neonatal intensive care unit, as well as an increased risk of neonatal mortality.

Congenital defects represent a significant diagnostic and therapeutic challenge in modern medicine and are often associated with prematurity, sometimes extreme. The overall mortality caused by complications of congenital defects increases when anomalies and prematurity coexist.^(12)^ Adam et al.^(13)^ demonstrated that newborns with major congenital anomaly are two-times more likely to have a preterm birth and a low birth weight.

Among cases of preterm birth, there was an increased frequency of congenital defects, since it includes a range of conditions associated with growth abnormalities and amniotic fluid alterations. Other potential factors are related to the defects themselves, such as anencephaly, a frequent cause of therapeutic prematurity (or provider-initiated), anomalies associated with polyhydramnios, and anomalies linked to significant intrauterine growth restriction, as seen in fetuses with chromosomal anomalies.^(12,13)^

Other issues that might be related to an increase in the prevalence of preterm birth in pregnancies affected by congenital anomalies as fetal conditions associated with an increased risk of stillbirth, such as hydrops, or conditions at risk of spontaneous preterm birth, more specifically, gastroschisis.^(14)^

The incidence of therapeutic preterm births in this study was quite significant, with more than one-third of newborns with anomaly being born preterm for therapeutic reasons, the vast majority via cesarean section. Despite the lack of evidence, cesarean delivery, whether elective or during labour, is common and may be influenced by institutional logistics, availability of neonatal intensive care beds, lack of a strong psychological structure of the women and families, and more severe defects, especially those with increased volume of the fetus. These factors may lead to the therapeutic anticipation of birth, which remains a controversial and unresolved topic, particularly regarding its impact on neonatal outcomes and its occurrence in low-resourced settings.

Amniotic fluid volume disturbances are also associated with preterm birth in fetuses with anomalies. Polyhydramnios occurs in pregnancies involving central nervous system and gastrointestinal tract anomalies, as well as hydrops, and is linked to contractility disorders and an increased risk of cesarean section. The association with spontaneous preterm birth is less clear and is likely restricted to severe cases of fluid volume increase, which are rarer and, therefore, more challenging to assess conclusively.^(15-17)^

In the main EMIP analysis, polyhydramnios was not associated with an increased risk of preterm birth;^(9)^ however, our analysis revealed a significantly higher incidence of polyhydramnios among fetuses with anomaly, with 100% prematurity in those with increased amniotic fluid volume. Although congenital anomalies are present in about one-third of polyhydramnios cases, with the potential for spontaneous labour and preterm rupture of membranes, a portion of preterm births is caused by other associated complications, such as diabetes, preeclampsia, multiple gestations, and fetal well-being alterations.

The significantly and expectedly worse perinatal outcomes in several aspects, including a sixfold higher neonatal mortality rate, reflect the elevated risks these newborns face and the need for specialised care with readily available human and material resources—factors that are crucial for prognosis and quality of life. Furthermore, early diagnosis and high-quality prenatal care allow for better treatment planning and preparation for the specific needs of newborns with congenital anomalies.

Congenital anomalies are currently the leading cause of neonatal mortality. Our study confirmed the poor prognosis of these newborns, with significantly higher morbidity and mortality rates, which can be attributed to numerous factors, including mainly prematurity. The interaction between prematurity, whether spontaneous or therapeutic and congenital anomalies accounts for approximately 32% of perinatal morbidity and 15% of perinatal mortality. These numbers are striking and raise concerns regarding the prevention of prematurity or the reduction of associated morbidity in these newborns. Some aspects warrant consideration: risk stratification and management in tertiary centres with multidisciplinary teams may be an important strategy, as the availability of resources, the experience of fetal medicine teams, pediatric surgeons, neurosurgeons, and neonatal intensive care specialists significantly impact survival rates and morbidity.^(12)^

Prematurity-related mortality varies drastically by geographic region, primarily due to differences in national income levels. In higher-income countries with higher Human Development Index (HDI) scores, neonatal mortality rates are significantly lower than in low and middle-income nations. For example, a newborn under 28 weeks has a 90% chance of death in the first few days of life in low-income countries, compared to only 10% in developed nations.^(1,5)^ This variation is attributable to multiple factors, including cultural, political, educational, and public health determinants. In addition, it is well known that fetal anomalies are earlier detected by ultrasound in high-income countries with less restrictive policies for abortion, thus also decreasing the corresponding perinatal mortality. Controlling well-established risk factors for prematurity, combined with systematic and minimally qualified prenatal and perinatal care for preterm newborns, is already sufficient to prevent thousands of deaths related to this condition.

Furthermore, the relationship between extreme nutritional status and preterm birth is well documented, affecting both obese and undernourished pregnant women. Routine periconceptional supplementation with folic acid and elemental iron, which has been standard practice in developed countries since at least the 1990s, is associated with a reduction in both isolated prematurity and prematurity associated with congenital anomalies, particularly neural tube defects (NTDs).

Other interventions related to the management of pregnant women in preterm labour or at risk of preterm birth can significantly alter neonatal outcomes, either by preventing preterm delivery or reducing the severe neonatal morbidity and mortality associated with it. According to WHO publications, corticosteroids for lung maturation alone save approximately 370,000 lives annually.^(1,5)^

Providing a heat source, early breastfeeding, and performing proper neonatal resuscitation save an additional 450,000 lives per year. Low-tech interventions, such as oxygen supplementation, magnesium sulfate for neuroprotection, and systematic antibiotic use in preterm premature rupture of membranes, also significantly impact preterm morbidity and mortality. ^(1,5)^

The economic expenditures associated with prematurity is high and increasing worldwide, especially when it is linked to fetal anomalies. A systematic review showed that antenatal corticosteroid are cost effective for preterm births, while tocolysis still needs a more detailed assessment.^(18)^ Preterm infants have a significantly higher impact in economic consequences due to the incidence of various health complications and developmental delays, with the occurrence being inversely proportional to gestational age at birth.^(19)^

Anderson et al. investigated the prevalence and outcomes of congenital heart diseases in very low birth weight preterm newborns in Brazil.^(20)^ Although the primary focus was on congenital heart diseases, the study highlights that the presence of a major congenital anomaly and a gestational age of less than 29 weeks are independently associated with increased neonatal mortality. This suggests that congenital anomalies, in general, may exacerbate the risks associated with extreme prematurity. An investigation of neonatal anthropometry of newborns with anomaly in a large South American population, including Brazil, showed that the prevalence of small-for-gestational-age newborns is higher among those with anomalies, both in term and preterm births.^(21)^ This indicates that congenital anomalies are associated with fetal growth restrictions, which may further complicate outcomes in cases of extreme prematurity. A population-based study assessing the Live Birth Information System (SINASC/DATASUS) from a city in southern Brazil demonstrated that congenital anomalies are associated with an increased likelihood of preterm birth (OR 3.18 [95%CI 2.14-4.74]).^(22)^ Tietzmann et al.^(23)^ addressed neonatal mortality in preterm newborns in southern Brazil, highlighting that congenital anomalies are one of the factors associated with high neonatal mortality rates in extremely preterm infants. Congenital anomalies were associated with a 2-fold risk for neonatal mortality in preterm newborns. (HR 2.82 [95%CI 2.29–3.48]). A multicenter study conducted in 10 referral maternity hospitals in Brazil reviewed causes of fetal deaths from the last 10 years. Congenital anomaly comprised approximately 15% of stillbirths.^(24)^

In summary, the literature suggests a significant association between congenital anomalies and extreme prematurity in Brazil, with important implications for neonatal mortality and morbidity. Congenital anomalies may increase the risk of complications in extremely preterm newborns, underscoring the need for specialised care and early intervention. For instance, congenital hydrocephalus presents a notable challenge, which requires extensive medical interventions such as shunt procedures, which are costly and resource-intensive.^(25)^ Neural tube defects, including anencephaly and spina bifida, also contribute significantly to neonatal morbidity and mortality. The implementation of folic acid fortification in Brazil has led to a reduction in the prevalence of these defects and associated healthcare costs, demonstrating the importance of preventive strategies in managing congenital anomalies.^(26)^ The distribution of healthcare resources for congenital anomalies is uneven across Brazil, with many health units lacking the necessary infrastructure for neonatal surgical care. This dispersion results in missed opportunities for quality and timely care, highlighting the need for better organisation and referral networks to optimise resource use and improve outcomes.^(27)^

Overall, the impact of congenital anomalies in Brazilian NICUs is substantial, necessitating enhanced healthcare infrastructure, early diagnosis, and preventive measures to improve neonatal outcomes and resource allocation. In addition, more information is needed regarding cases of anomalies early detected during pregnancy that were electively interrupted, even if some of them are obviously illegally performed.

This study has the strength of being one of the first to approach fetal anomaly in preterm births in a multicenter Brazilian study. However, it is limited by using data from reference services in Brazil that are part of a national network for studies of reproductive and neonatal health. This potentially overestimates the prevalence of severe cases and more complex anomalies, which may not fully represent the reality of smaller facilities; consequently, care should be taken when generalising the results. Additionally, the questionnaire did not allow for detailed information on specific anomalies and focused on major anomalies, while minor or late-onset anomalies may not have been detected. Also, diagnosis depended on local clinical characteristics, which could vary in sensitivity across the different participating centres, due to logistical or resource constraints.

Our data, however, underscores the association between congenital anomalies and preterm birth, as well as their repercussions on neonatal outcomes. This highlights the need to refine antenatal care by investing in specialised neonatal support and standardising clinical protocols to determine the timing and mode of delivery. Moreover, promoting measures to prevent anomalies (folic acid supplementation) and consolidating policies for centralised management of complex cases in tertiary centres are crucial to minimising the morbidity and mortality associated with these dual risk factors.

## Conclusion

There was an association between preterm birth and congenital anomalies, especially between 28 and 36 weeks. The most prevalent anomalies involved the cardiovascular system, nervous system, and abdominal wall. Perinatal outcomes were worse among preterm newborns with anomalies, with higher rates of mortality and prolonged hospitalisation in neonatal intensive care units compared with term.

## Data Availability

The research data are described in the article presented.
